# Targeting Systemic Sclerosis from Pathogenic Mechanisms to Clinical Manifestations: Why IL-6?

**DOI:** 10.3390/biomedicines10020318

**Published:** 2022-01-29

**Authors:** Anca Cardoneanu, Alexandra Maria Burlui, Luana Andreea Macovei, Ioana Bratoiu, Patricia Richter, Elena Rezus

**Affiliations:** 1 Department of Rheumatology, University of Medicine and Pharmacy “Grigore T Popa”, 700115 Iasi, Romania; maria-alexandra.burlui@umfiasi.ro (A.M.B.); luana.macovei@umfiasi.ro (L.A.M.); ioana.bratoiu@umfiasi.ro (I.B.); patricia.richter@umfiasi.ro (P.R.); elena.rezus@umfiasi.ro (E.R.); 2 Rehabilitation Hospital, 700661 Iasi, Romania

**Keywords:** systemic sclerosis, interleukin-6, Tocilizumab, pulmonary fibrosis

## Abstract

Systemic sclerosis (SS) is a chronic autoimmune disorder, which has both cutaneous and systemic clinical manifestations. The disease pathogenesis includes a triad of manifestations, such as vasculopathy, autoimmunity, and fibrosis. Interleukin-6 (IL-6) has a special role in SS development, both in vascular damage and in the development of fibrosis. In the early stages, IL-6 participates in vascular endothelial activation and apoptosis, leading to the release of damage-associated molecular patterns (DAMPs), which maintain inflammation and autoimmunity. Moreover, IL-6 plays an important role in the development of fibrotic changes by mediating the transformation of fibroblasts into myofibroblasts. All of these are associated with disabling clinical manifestations, such as skin thickening, pulmonary fibrosis, pulmonary arterial hypertension (PAH), heart failure, and dysphagia. Tocilizumab is a humanized monoclonal antibody that inhibits IL-6 by binding to the specific receptor, thus preventing its proinflammatory and fibrotic actions. Anti-IL-6 therapy with Tocilizumab is a new hope for SS patients, with data from clinical trials supporting the favorable effect, especially on skin and lung damage.

## 1. Introduction

Systemic sclerosis (SS) is a chronic autoimmune disease that has both cutaneous and systemic clinical manifestations, with the latter being associated with high disease morbidity [1]. The pathogenic mechanisms of this condition are not yet fully understood. However, it is known that the pathogenesis of the disease is composed of a triad: vasculopathy, autoimmunity, and fibrosis [2]. All these mechanisms are interconnected, with each having a particular role in the occurrence of clinical manifestations and disease progression. Immunological mechanisms include both innate and adaptative immunity and involve many cells, such as: B and T lymphocytes, mast cells, macrophages, and dendritic cells [3]. Vasculopathy is characterized by damage and endothelial activation and participates in the initiation and perpetuation of autoimmunity [4]. Activation of fibroblasts and excess deposition of extracellular matrix causes the appearance of fibrosis, which is also related to autoimmunity and vasculopathy [3].

**Figure 1:**
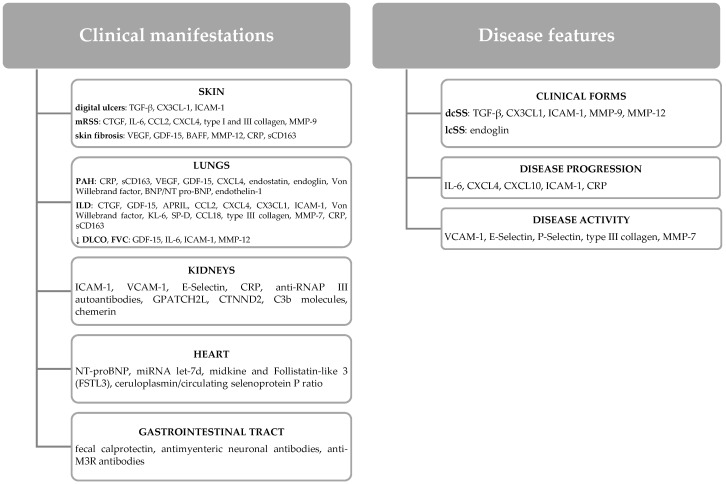
Potential biomarkers of organ involvement and disease features in SS. Key: TGF: transforming growth factor; GDF-15: growth differentiation factor 15; BAFF: B-cell-activating factor belonging to the tumor necrosis factor family; APRIL: a proliferation-inducing ligand; MMP: matrix metalloproteinases; BNP: brain natriuretic peptide; NT-proBNP: N-terminal-pro hormone BNP; CTG: connective tissue growth factor; mRSS: modified Rodnan total skin thickness score; ILD: interstitial lung disease; IL-6: interleukin 6; DLCO: diffusing capacity of carbon monoxide; PAH: pulmonary arterial hypertension; ICAM-1: intercellular adhesion molecule 1; dcSSc: diffuse cutaneous systemic sclerosis; VEGF: vascular endothelial growth factor; lcSSc: limited cutaneous systemic sclerosis; KL-6: krebs von den Lungen-6; SP-D: surfactant protein-D; CCL2: monocyte chemoattractant protein-1; CXCL4: platelet factor 4; VCAM-1: vascular cell adhesion molecule-1; CRP: C-reactive protein; sCD163: soluble CD163; anti-RNAP III antibodies: anti-RNA polymerase III antibodies; anti-M3R antibodies: anti-human muscarinic receptor M3 antibodies; miRNA let-7d: micro RNA let-7d; GPATCH2L: G-patch domain-containing protein 2-like; CTNND2: catenin delta 2.

In the early stage of the disease, an inflammatory profile was highlighted, characterized by an increased secretion of proinflammatory cytokines, with the predominant immune response being Th1 and Th17 cells [5]. Moreover, innate immunity, by activating NK/NKT-like cells, plays a decisive role in this initial stage of the disease [6]. The late stage of SS has a predominant Th2-type immune response and is defined by the presence of fibrotic changes [5]. Knowing these important data involved in the pathogenesis of the disease, numerous studies have correlated the clinical manifestations with the excessive secretion of some molecules, with the latter being considered potential biomarkers of the disease [7] (Figure 1).

Given the complex pathogenic mechanisms and the multitude of cells and cytokines, we will continue to focus on interleukin 6 (IL-6), which is considered to be one of the major fibrogenic cytokines along with interleukin 4 (IL-4) and transforming growth factor (TGF)-β [3].

## 2. IL-6: General Data

IL-6 is part of a large family of cytokines, having important roles in regulating immunity, hematopoiesis, inflammation, and oncogenesis. In the past, depending on its biological effect, IL-6 had different names: B lymphocyte-stimulating factor 2 (BSF-2), hepatocyte-stimulating factor (HSF), or interferon-β2 [8]. In 1989, it was found that all these molecules are identical, so it was renamed IL-6, a name used until today [9].

The biological effect of IL-6 can be achieved by two signaling pathways: the classical pathway, which uses a membrane receptor, and the trans-signaling pathway, which involves the presence of a soluble IL-6 receptor. The classical signaling pathway involves the binding of IL-6 to the membrane receptor-IL-6R [10]. Normally, the expression of this receptor is decreased, being found in several cells, especially hepatocytes and some immune cells [11,12]. IL-6R has no intrinsic signaling capacity. The IL-6/IL-6R interaction induces the dimerization of an IL-6R signaling-related cytokine called gp130 [13,14,15]. Unlike IL-6R, this gp130 molecule is ubiquitously expressed and has an intrinsic signaling capacity. The IL-6/IL-6R complex bound to gp130 initiates cellular signaling via several intracellular or extracellular pathways [10,12,16]. Gp130-mediated activation of Janus kinase (Jak) family members and subsequent phosphorylation of specific tyrosine residues cause phosphorylation and dimerization of STAT (signal transducer and activator of transcription proteins) [10,16]. The latter determines the transcriptional activation of IL-6-dependent genes [16].

The alternative IL-6 trans-signaling pathway involves the presence of a soluble receptor (sIL-6R) [16,17,18,19]. This receptor is formed after cleavage of IL-6R by activated ADAM17 (Disintegrin Metalloproteases 17) and ADAM10 (Disintegrin Metalloproteases 10) [20,21]. Binding of IL-6 to sIL-6R and subsequently to gp130 IL-6/sIL-6R determines intracellular signaling, which is very similar to classical IL-6R signaling. In addition, sIL-6R has the advantage of binding circulating IL-6, thereby prolonging the half-life of IL-6 [22]. Moreover, sIL-6R participates in the interaction between leukocytes and vascular endothelium, causing the endothelial production of MCP-1 (monocyte chemoattractant protein-1), a key chemokine that regulates monocyte/macrophage infiltration of the vascular wall [23].

To limit the proinflammatory effects of IL-6, IL-6 signaling is regulated by several inhibitory molecules and processes. Inhibition of IL-6 signaling is important in order to limit inflammation and its side effects, such as extensive tissue damage. Thus, STAT3-dependent transcription is rapidly inactivated by the cytokine 3 signaling suppressor (SOCS3) [24,25,26,27]. IL-6-induced SOCS3 expression acts as a major negative feedback loop for IL-6 signaling. Another inhibitory mechanism, a soluble form of gp130 (sgp130), has been identified in patients’ plasma [28,29]. sgp130 serves as a functional antagonist of IL-6 trans-signaling by binding to the IL-6/sIL-6R complex and preventing IL-6/IL-6R complex binding to the gp130 membrane molecule [30]. A third mechanism of inhibition of IL-6 signaling involves a STAT-3 inhibitor (PIAS3-Protein Inhibitor of Activated STAT 3) that blocks the interaction between phosphorylated STAT3 and cellular DNA and finally genetic transcription [31].

**Figure 2:**
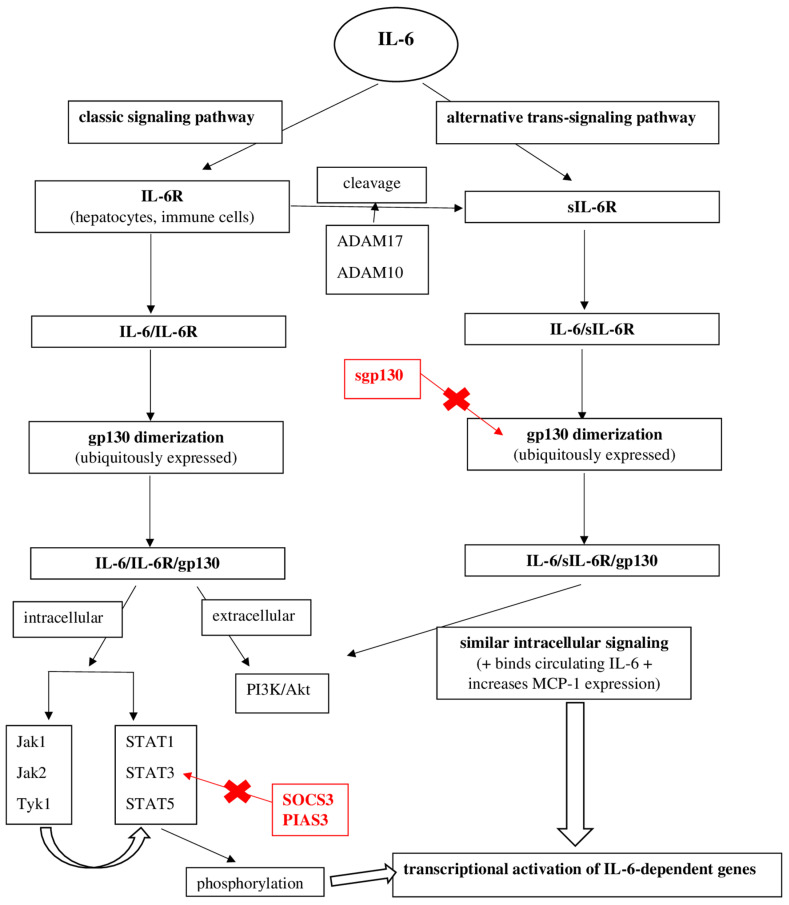
The intracellular signaling pathways of IL-6 and the main negative feedback loops that block the production of its biological effects. Key: IL-6: interleukin-6; IL-6R: interleukin-6 receptor; sIL-6R: soluble interleukin-6 receptor; gp130: glycoprotein 130; Jak: janus kinase; STAT: signal transducer and activator of transcription proteins; SOCS3: cytokine 3 signaling suppressor; PIAS3: protein inhibitor of activated STAT 3; ADAM: disintegrin metalloproteases; MCP: monocyte chemoattractant protein-1.

Figure 2 schematically shows these two intracellular signaling pathways of IL-6, and the main negative feedback loops that block the production of its biological effects (Figure 2).

IL-6 is a key cytokine in inflammation, having a systemic polymorphic pathway. Thereby, after the initial stage of inflammation and local production, IL-6 is transported through the blood vessels in the liver. Here, it determines the formation and increased secretion of acute phase proteins, such as C-reactive protein (CRP), serum amyloid A, fibrinogen, and hepcidin. On the other hand, IL-6 has an inhibitory effect on the secretion of fibronectin, albumin, and transferrin [32,33]. Due to the stimulatory effect of hepcidin formation, the iron transporter is blocked in the gut (ferroportin 1) [34]. Thus, the IL-6-hepcidin axis is responsible for hyposideremia and anemia associated with chronic inflammation. In addition, IL-6 has the ability to promote zinc deposition in hepatocytes by increasing the expression of ZIP14-specific transporter. The regulation of ZIP14 by IL-6 contributes to hepatic accumulation of zinc and to the decrease in the serum zinc concentration highlighted in inflammation [32,35].

IL-6 plays an important role in the innate or adaptative immune response by stimulating CD4+ T cell differentiation [32]. Data from the literature support the indispensable role of the IL-6-TGF-β axis in differentiating Th17 cells from naive T CD4+ cells [36]. Moreover, inhibition of regulatory T cell differentiation (Treg) occurs [37]. This Th17/Treg imbalance is responsible for disrupting immune tolerance, leading to chronic inflammatory autoimmune disorders [38]. IL-6 has the ability to stimulate follicular T helper cell differentiation and the production of IL-21, which is responsible for the synthesis of immunoglobulins (Ig), especially IgG4 [39]. IL-6 can induce CD8+ cell differentiation into cytotoxic T cells [40]. IL-6 also acts on B lymphocytes, activating and transforming them into plasma cells, thus promoting the production of autoantibodies and hypergammaglobulinemia [32].

IL-6 can have a direct effect on osteoblast precursors, blocking their differentiation and maturation [41]. IL-6 induces the secretion of matrix metalloproteinases (MMP-1, MMP-3, MMP-13) from chondrocytes and synovial cells. MMP-1 and MMP-13 are able to break type II collagen, and MMP-3 exerts its action on the extracellular matrix, fibronectin, and laminin [42]. In addition, elevated levels of IL-6 in bone marrow stromal cells stimulate the activator receptor of nuclear factor kappa-B ligand (RANKL), leading to osteoclast differentiation and activation [43].

**Figure 3:**
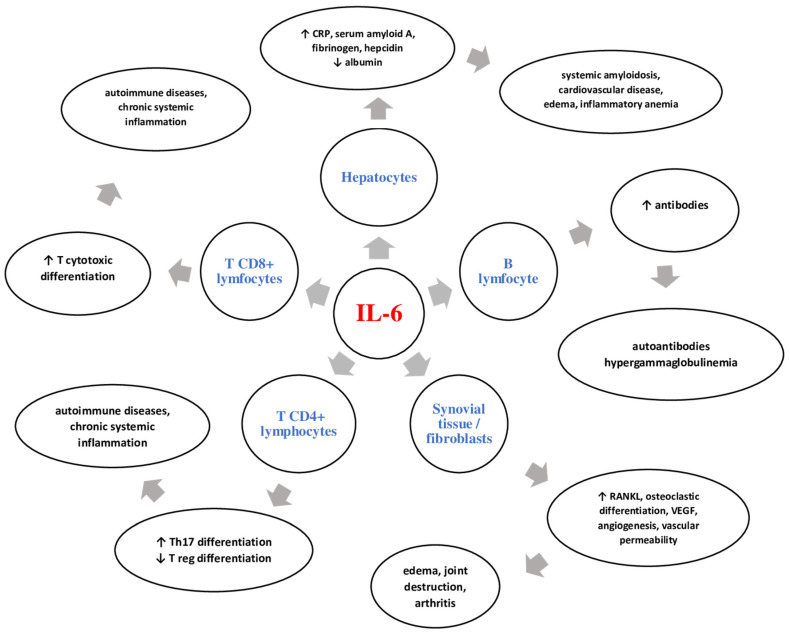
The action of IL-6 on various cells and its local and systemic manifestations. Key: IL-6: interleukin 6; CD: cluster of differentiation; CRP: C-reactive protein; RANKL: activator receptor of nuclear factor kappa-B ligand; VEGF: vascular endothelial growth factor; Treg: regulatory T cells.

The action of IL-6 is systemic, being a key element in the development and perpetuation of inflammation. IL-6 participates in the formation of a cytokine storm by activating immune cells and stimulating the secretion of new inflammatory mediators. Figure 3 shows the main cells on which IL-6 acts and the systemic effects it can have.

## 3. IL-6 in Systemic Sclerosis

IL-6 has a particular role in the pathogenesis of SS, both in vascular damage and in the development of fibrosis. The increase in collagen production is achieved through different pathways, such as differentiation of myofibroblasts, inhibition of the secretion of matrix metalloproteinases with collagenolytic effect, and activation of fibroblasts [44]. The trans-signaling pathway is mandatory for collagen production and for the appearance of fibrotic changes due to the presence of sIL-6R [45]. This is supported by data from murine studies. The occurrence of an SS-like condition with pulmonary fibrosis and skin thickening was determined by IL-6 signaling [46]. On the other hand, a decrease in pulmonary fibrosis and inflammation was observed in IL-6-deficient mice [47].

Genetic susceptibility plays an important role in the pathogenesis of SS; therefore, the polymorphism of the IL-6 gene has been studied in various clinical trials. The most studied gene is *rs1800795* (also known as −*174 C/G*), with G alleles being associated with increased IL-6 levels [48,49]. An analysis of −*174 C/G* IL-6 polymorphism in a cohort of 102 SS patients revealed increased IL-6 gene expression in G-allele carriers. This genetic polymorphism of IL-6 was correlated only with the presence of gastrointestinal manifestations, not with skin and lung damage [50]. Other data obtained after studying 20 cases with SS support the link between IL-6 polymorphism, especially the homozygous *GG* form, and an active and disabling disease [51].

### 3.1. IL-6 and SS Features

Elevated serum and skin levels of IL-6 have been highlighted in both early and late stages of SS [52,53]. In the early stages, IL-6 participates in vascular endothelial activation and apoptosis [54], leading to the release of damage-associated molecular patterns (DAMPs), which maintain inflammation and autoimmunity [55].

Numerous data support this increase in IL-6 levels, both in the tissues and in the serum of patients with SS. Feghali’s study showed elevated levels of IL-6 in the skin tissue supernatant cultures of SS patients, even 30 times higher than the control arm [56]. By culturing peripheral blood mononuclear cells isolated from cases of SS with type I collagen, Gurram highlighted an increased level of IL-6 in the supernatant, which shows that IL-6 can be highly secreted by other unaffected tissues [57]. Various methods for measuring serum IL-6 levels have been used over time, such as bioassays and ELISA (enzyme-linked immunosorbent assay). Needleman, using a bioassay method, found detectable concentrations of IL-6 in the serum of SS patients [58]. The ELISA method provided important data on elevated IL-6 levels in these patients, levels that correlated with the degree of skin damage quantified by the cutaneous score [59,60].

It seems that the concentration of IL-6 depends on the duration and form of SS. Thus, a study that included 55 SS patients analyzed the levels of IL-6, sIL-6R, and sgp130 according to the form of the disease. Patients were divided into 4 arms: 12 having an lcSS (limited cutaneous SS) and an early form of disease (less than 3 years from disease onset), 22 with lcSS and a late form (over 3 years from disease onset), 9 cases of an early dcSS form (diffuse cutaneous SS), and 12 cases of a late dcSS form [61]. Following the statistical analysis, the authors found significantly higher levels of IL-6 in the early SS form compared to the control arm, with high levels found especially in patients with pulmonary fibrosis. IL-6 was inversely correlated with vital lung capacity (VPC). IL-6 levels have also been associated with acute phase reactants and A and G immunoglobulin levels [61]. Regarding sIL-6R, elevated levels were observed in a limited SS form compared to the control. For sgp130, no statistically significant data were highlighted [61].

The fact that there is a different secretion of cytokines depending on the stage of the disease has been demonstrated by clinical trials. Thus, Matsushita showed a significant increase in IL-6 and IL-10 in the early stages of the disease, and a significant numerical decrease after 6 years of evolution. Opposite data have been described for IL-12, whose values were decreased in the early stages and more than 15 times increased in the disease with a 6-year evolution [62]. The level of IL-6 has been shown to be inversely proportional to that of IL-13, with early-stage patients having a high IL-6 and low IL-13 amount [63].

### 3.2. IL-6 and Skin Manifestations

IL-6 plays an important role in the development of fibrotic changes by mediating the transformation of fibroblasts into myofibroblasts, with the latter producing an excessive amount of collagen that infiltrates various organs and tissues, including the heart [64]. Myofibroblasts were found in the dermis in the early and progressive SS forms, with histological analysis indicating their disappearance in late (atrophic) forms of the disease [65]. Furthermore, a study that included a model of bleomycin SS model mice showed a numerical decrease in myofibroblasts after administration of anti-IL-6R antibodies [66]. The same data were highlighted by Kawaguchi, who performed cultures of cutaneous fibroblasts isolated from SS patients and found a high level of procollagen type I in the supernatant. After incubating these cultures with anti-IL-6R antibodies, a numerical decrease in collagen fibers was observed [67].

### 3.3. IL-6 and Lung Manifestations

The involvement of IL-6 in fibrotic lesions has been proven by clinical trials, especially in lung damage. Pulmonary fibrosis in SS has a multifactorial etiopathogenesis that includes both infiltration with inflammatory cells at the alveolar, peribronchiolar, and interstitium levels, and an intense and aberrant proliferation of fibroblasts that cause the deposition of fibronectin, type I and III collagen fibers, and tenascin [68,69].

Regarding IL-6, Crestani’s data included the analysis of cultures of alveolar macrophages obtained after bronchoalveolar lavage in SS patients. In the supernatant, the researchers found an increased level of IL-6 compared to the control group [70]. Other recently published data point to the same increased level of IL-6 in cases of SS with advanced pulmonary fibrosis due to the perpetuation of chronic inflammation, inhibition of the secretion of metalloproteinases, and increased collagen fiber synthesis [71]. On the other hand, another important pathogenic mechanism involved in the development of pulmonary fibrosis is the enhancement of IL-6 trans-signaling via ADAM-17 by activated macrophages, which increases the extracellular matrix deposits and the proliferation of fibroblasts [72]. In this situation, it seems that inhibition of the IL-6 trans-signaling pathway may be a useful therapeutic approach [71,72].

IL-6 levels, especially in early SS with mild forms of pulmonary fibrosis, appear to have a prognostic value for impaired lung function and increased mortality. This is supported by the analysis of 74 cases of SS associated with pulmonary fibrosis. An IL-6 value of more than 7.67 pg/mL can be considered a predictor of decreased DLCO (diffusing capacity of lung for carbon monoxide) and FVC (forced vital capacity) in the first year. It also correlates with an increase in mortality in the first 30 months of the disease [73]. Other data obtained after analyzing a cohort of 68 SS patients support the prognostic value of IL-6, with increased values being correlated with the extension of skin involvement at the 3-year follow-up, the development of pulmonary fibrosis, and worse long-term survival. Therefore, IL-6 can be considered a predictive marker for disease progression [60,74]. Furthermore, IL-6 levels may be useful in stratifying patients regarding disease activity and survival outcome [75,76,77].

### 3.4. IL-6 and Cardiovascular Manifestations

IL-6 plays an important role in the cardiovascular involvement of SS. In this regard, an analysis of 20 cases with SS showed positive correlations between the level of IL-6 and the disease duration; EUSTAR (European Scleroderma Study Group) activity score; musculoskeletal, vascular, and respiratory status; and pressure peak in the pulmonary artery [78]. Other positive associations were noted with the pulmonary fibrosis score quantified by HRCT (high-resolution computer tomograph). Negative correlations were scored between IL-6, DLCO, the 6-min walk distance, and right ventricle systolic function parameters [78].

Furthermore, IL-6 may be considered a marker for pulmonary arterial hypertension (PAH) [79]. PAH mainly occurs in the limited form of the disease [80] and is closely related to the proliferation of endothelial cells and the formation of extracellular matrix deposits that cause intimate thickening of the capillaries and pulmonary arterioles [81]. Systemic inflammation, represented by IL-6, plays an important role in the development of PAH due to perivascular inflammatory cell infiltrates [81]. The risk of death is higher compared to idiopathic PAH [82,83], being linked to an accentuated intimal hyperplasia, fewer plexiform lesions, and the involvement of pulmonary veins [81,84].

### 3.5. IL-6 and Gastrointestinal Manifestations

In addition to lung damage, 90% of SS patients also have gastrointestinal involvement (GI) [85], which is the 3rd leading cause of death [86]. GI impairment is associated with a marked disability and a decreased quality of life in SS patients, leading to reduced survival [86,87]. IL-6 gene polymorphism increases disease susceptibility, being associated with different clinical manifestations [49]. Zekovic’s study, which included 102 SS cases, analyzed the correlations between IL-6 expression and GI manifestations and the presence of a specific disease genotype /phenotype [50]. The results showed that the presence of the C-allele correlates with increased IL-6 gene expression and with GI manifestations, with the most common being abdominal distension and worsening of the GI score (UCLA GIT 2.0) [50].

The pathogenic mechanisms involved in the occurrence of GI manifestations are complex and include concentric intimate thickening and deposition of collagen and mucoid molecules [88], adventitia fibrosis [89], autonomic dysfunction affecting neuromuscular junction, smooth muscle atrophy [90,91], presence of antimyenteric neuronal antibodies, anti-U3 RNP and anti-muscarinic-3 receptor antibodies [92,93,94,95], and small intestinal bacterial overgrowth serologically expressed by an increased fecal calprotectin [96]. The clinical manifestations are polymorphic and may include the entire GI tract, with patients complaining of dysphagia, intestinal transit disorders, fecal incontinence, pseudo-obstruction, or having clinical signs of malabsorption [97].

### 3.6. IL-6 and Kidney Manifestations

SS kidney disease has an incidence of up to 10% [98] and is associated with increased long-term mortality [99], with scleroderma renal crisis (SRC) being a medical emergency. Genetic predisposition, autoimmune mechanisms, inflammatory cascade, and vasculopathy are the main mechanisms involved in the pathogenesis of SRC. An increased expression of two proteins: GPATCH2L and CTNND2, was highlighted in a recent study that analyzed kidney biopsy pieces in SS patients, suggesting the involvement of genetic susceptibility in the development of the disease [100]. Among autoantibodies, anti-RNA polymerase III antibodies are associated with renal impairment and have a prognostic value [101]. The main inflammatory markers involved in kidney damage are interleukins (IL-2r, IL-6, IL-10, IL-18) and chemokines, such as MCP-1 (monocyte chemoattractant protein-1) [102,103]. Vasculopathy refers to intimal proliferation and thickening of the renal arcuate and interlobular arteries, which causes activation of the renin-angiotensin system [104,105].

Clinically, patients present with rapidly progressive acute renal failure, malignant hypertension that may be complicated by hypertensive encephalopathy and retinopathy, pulmonary edema, or seizures. Microangiopathic hemolytic anemia, cardiac arrhythmias, myocarditis, and fever may also occur [106,107].

**Figure 4:**
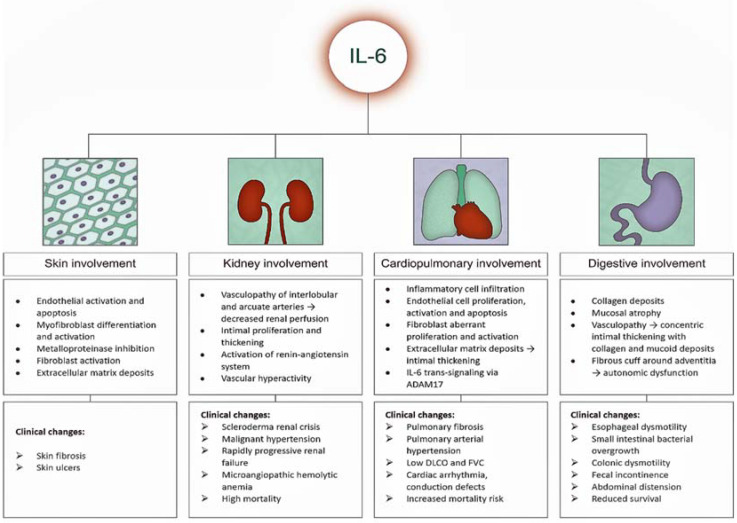
Systemic harmful effects of IL-6 on the skin, cardiopulmonary, gastrointestinal and renal system. Key: IL-6, interleukin-6; ADAM17: disintegrin metalloproteases 17; PAH: pulmonary arterial hypertension; DLCO: diffusing capacity of lung for carbon monoxide; FVC: forced vital capacity.

All of these systemic harmful effects of IL-6 are shown schematically in Figure 4.

## 4. Targeting IL-6 in Systemic Sclerosis

Given the above data, we can sustain that IL-6 can be considered a key cytokine in the development and evolution of SS. For these reasons, anti-IL-6 therapy with Tocilizumab is a new hope for SS patients, with data from clinical trials supporting both cutaneous and pulmonary improvement. Tocilizumab is a humanized monoclonal antibody that inhibits IL-6 binding to mIL-6R (membrane IL-6R) and sIL-6R and prevents its pro-inflammatory and fibrotic effects [108]. In addition to SS, this beneficial effect has been shown in a variety of conditions, such as rheumatoid arthritis, juvenile idiopathic arthritis, Takayasu’s arteritis, Still’s adult disease, giant cell arteritis, and Castleman’s disease [109,110,111,112,113].

The benefits of Tocilizumab in SS have been highlighted in phase 2 and 3 studies since 2016. The FaSScinated study is a randomized, double-blind, placebo-controlled phase 2 trial that included 87 SS patients from 35 sites [114]. Randomization was 1: 1, with 43 cases in the SS group for whom the onset of the disease was less than 5 years and who received subcutaneous Tocilizumab 162 mg weekly versus a placebo arm, which included 44 patients. No patient had been on background immunosuppressive therapy. The primary endpoint was an improvement in the modified RODNAN skin score (mRSS) at 24 weeks, which was not achieved, but the data showed a cutaneous improvement after Tocilizumab administration (mRSS decrease with 3.92 for Tocilizumab vs. 1.22 for placebo, *p* = 0.0915). A more significant skin improvement was seen after 48 weeks with Tocilizumab, but this still did not reach statistical significance (mRSS decrease with 6.33 for Tocilizumab vs. 2.77 for placebo, *p* = 0.0579). Data regarding lung damage were promising, showing a significantly smaller decrease in FVC for Tocilizumab at week 48 compared with the placebo (*p* = 0.0373). Regarding fatigue, itching, clinician global disease severity, and disability, there were no differences between the two groups. The safety of Tocilizumab was comparable to the placebo (42 vs. 40), but more severe infections were reported in the active arm (7 vs. 2), with 1 death. The authors concluded that, although the skin primary endpoint was not achieved, the decrease in lung involvement was significant, thus opening new research perspectives.

Two years later, Khanna published the results of a phase 3 study (focuSSced study) that focused on Tocilizumab’s effectiveness on skin and lung fibrosis, also including data regarding disability, treatment failure, and safety [115]. The randomized, double-blind, placebo-controlled trial included 210 patients with an early dcSS (<5 years) without background immunosuppressive treatment. Randomization was 1: 1, with 104 cases receiving subcutaneous Tocilizumab 162 mg weekly and 106 on placebo. The primary endpoint was an improvement in the mRRS skin score at week 48, which was not achieved (*p* = 0.10). Secondary objectives included the change in the predicted percentage of FVC, patient- and physician-reported outcomes, and time to treatment failure at week 48. Significant data were obtained for the predicted percentage of FVC, with Tocilizumab being effective in preserving lung functionality (*p* = 0.002). The results on disability and the overall physician and patient assessment of the disease were not significant. The Tocilizumab safety profile was good, with the most common side effects being infections.

Other recently published data support Tocilizumab’s beneficial effect on skin fibrosis. A study on the effect of IL-6 blockade on the molecular, genomic, and functional characteristics of cultured fibroblasts from skin biopsies of SS patients was performed [116]. The analysis included 12 patients treated with Tocilizumab or placebo for 24 weeks. An activated molecular and functional phenotype was maintained after 24 weeks with placebo. In contrast, fibroblasts in Tocilizumab patient cultures underwent beneficial changes, such as decreased migration, proliferation and contraction, and low collagen fiber production. Furthermore, by analyzing the genetic profile dominated by genes that promote fibrosis (TGFβ-regulated genes, COL1A1), it was found that Tocilizumab can induce normalization of the genetic phenotype, thereby improving skin thickening [116]. The findings of this analysis were promising and highlighted the dual role of IL-6 blockade in inflammation and fibrosis.

Other data on the improvement of cutaneous fibrosis with Tocilizumab come from smaller studies, even from case reports and case series, considering the low incidence of the disease. Shima followed 2 patients with SS refractory to conventional therapies who received Tocilizumab 8 mg/kg/month for 6 months. Skin damage was assessed by both the mRRS score and with a Vesmeter device, which measures viscosity, elasticity, and skin thickening [117]. Tocilizumab therapy resulted in skin softening quantified by both scores. Another study by Shima included seven SS cases; its main purpose was to investigate the cutaneous efficacy of Tocilizumab quantified by the mRRS score and to analyze the factors that contribute to treatment response [63]. Skin improvement was evident after Tocilizumab therapy, but the data were not statistically consistent. Moreover, the decrease in the mRSS score was higher in cases with a significant inflammatory syndrome (quantified by CRP) and shorter disease duration. Negative correlations were found between the mRRS score and chemokine CCL-5 and IL-13 levels.

In addition to improving skin fibrosis, Tocilizumab appears to be effective in osteoarticular changes. Zacay et al. administered Tocilizumab in 16 cases of SS without clinical response to other immunosuppressants. All patients had musculoskeletal involvement. Twelve patients with arthritis/ arthralgia showed a significant improvement after treatment. One case of myalgia improved, and three out of four with myositis showed normalization of muscle enzymes after Tocilizumab [118]. In addition, the authors found a significant improvement in the mRSS skin score, which decreased by 11 points, and an improvement in lung capacity in 46% of cases. All these favorable responses were mainly recorded in patients with an early disease. The same positive effect on musculoskeletal manifestations is supported by EUSTAR (The European Scleroderma Trials and Research group), which analyzed the response to Tocilizumab and abatacept in a cohort of 27 SS cases. All patients with polyarthritis significantly improved after 5 months of therapy. However, the mRSS score did not show a statistically significant decrease [119]. In the two cases presented by Shima [117], one of the patients had a significantly limited range of joint motion, which improved after Tocilizumab therapy, thus strengthening the efficacy of the IL-6 inhibitor on joint involvement and supporting its inclusion in the SS treatment algorithm [120].

Regarding lung damage, one of the most important complications of the disease and the leading cause of death, Tocilizumab therapy is a recently approved solution linked to decreased lung worsening and maintenance of pulmonary function. The results of the extension of the phase 3 focuSSced study support this pulmonary improvement after switching from placebo to Tocilizumab [121]. Thus, 78 patients in the placebo arm switched to Tocilizumab. After 2 years of treatment, 50% of them maintained a constant level of FVC. Only 3% (2 cases) developed an increase in pulmonary fibrosis quantified by a decrease in FVC of over 10%. All of these patients showed not only maintenance of lung capacity, but even an improvement in FVC of 0.6% after 2 years. The results were similar to those in patients treated with Tocilizumab from the beginning. Moreover, the 48-week extension of the faSScinate study strengthened the effect of Tocilizumab therapy in stabilizing lung function. In addition, there was a further improvement in the mRSS score at week 96 [122].

Recently, in 2021, a post hoc analysis of the focuSSced trial was published, which aimed to analyze the preservation of lung function in 136 cases of SS and intestinal lung disease. As a novelty, stratification of patients was conducted according to the degree of pulmonary fibrosis: mild (5–10%), moderate (10–20%), and severe (>20%) [123]. The included patients had an early SS with progressive skin disease and pulmonary fibrosis, so they were in the immunoinflammatory phase of the disease. Moderate and severe fibrotic lung impairment had an increased incidence of 77%. The authors identified inverse correlations between the degree of pulmonary fibrosis and the predicted FVC percentage. FVC stabilization was similar in all study arms regardless of fibrosis and was significantly more important for Tocilizumab compared to the placebo (−0.1% Tocilizumab vs. −6.3% placebo, *p* < 0.0001). Because Tocilizumab’s favorable pulmonary effects were observed in the early fibrotic phase of SS, the authors hypothesized that a window of therapeutic opportunity exists during this stage.

Tocilizumab may be considered as a rescue therapy in non-responsive cases. Narvaez included in an analysis nine cases of progressive SS with interstitial lung disease refractory to corticosteroid therapy (low and medium doses), rituximab, and other immunosuppressants (cyclophosphamide, azathioprine, mycophenolate mofetil). Patients received Tocilizumab (both intravenously and subcutaneously) in combination with mycophenolate mofetil for 6 months; prednisone below 5 mg/daily was used in 7 cases. The results regarding lung damage were promising, with 44% of patients experiencing both stabilization and an improvement in lung capacity quantified by FVC, DLCO, and the 6-min walking test [124].

Another recent analysis, reinforced by real-world data, indicated again the beneficial effect of Tocilizumab on skin and lung damage, its long-term safety, and an improvement in patient-reported outcomes. Thus, Khanna’s study of 82 SS cases showed an improvement in mRSS and FVC and a good safety profile after 96 weeks of Tocilizumab administration [125]. Panopoulos’ analysis of 21 SS patients receiving subcutaneous Tocilizumab for 1 year showed the same efficacy data on the mRSS score and polyarticular impairment, also providing stabilization of lung damage and an improvement in patient-reported outcomes [126].

The benefit of Tocilizumab therapy in SS heart disease has been less studied so far. Due to repeated lesions of ischemia and reperfusion, histological changes in band-like necrosis, and diffuse macular fibrosis, SS can be considered an independent risk factor for cardiovascular disease [127,128]. This year, the case of a young patient diagnosed with lcSS in 2015 who developed ventricular extrasystoles and changes in cardiac reperfusion highlighted on an interventricular septum biopsy test was published. Mycophenolate mofetil 2 g daily was administered for 3 months, but the heart condition worsened. Intravenous treatment with Tocilizumab 8 mg/kg/monthly was initiated with favorable results regarding clinical symptoms and reperfusion defects [129].

Finally, there are data indicating the favorable effect of Tocilizumab on SS juvenile forms (JSS). The first published study of the efficacy of Tocilizumab in JSS was published last year and included nine JSS cases on anti-IL-6R therapy in combination with methotrexate, mycophenolate mofetil, or corticosteroids. Patients mainly had pulmonary and gastrointestinal manifestations [130]. After a mean follow-up of 24 months, statistically significant data were recorded for skin damage (decreased mRSS), lung function (increased DLCO), global patient assessment (PGA), and Juvenile Systemic Sclerosis Severity (J4S), which improved.

All these data presented above regarding the clinical efficacy of Tocilizumab therapy in SS patients are summarized in Table 1.

## 5. Conclusions

Although we have a rich therapeutic arsenal, SS remains a serious condition that involves a multidisciplinary approach and targeted therapies. The pathogenesis of the disease is complex and multifactorial, and is still incompletely known. IL-6 has an important role in both vasculopathy and fibrosis and is associated with various clinical manifestations. Blocking IL-6R with Tocilizumab results in many clinical and biological benefits due to its anti-inflammatory and anti-fibrotic effects. Recent studies have opened the way for Tocilizumab in SS, supporting its efficacy and safety.

## Figures and Tables

**Table 1:** Data regarding the clinical efficacy of Tocilizumab therapy in SS patients.

Author	Number of Patients	Clinical and Paraclinical Parameters	Follow-Up Period	Results
Shima et al., 2010 [117]	2	mRSS	6 months	skin thickening improvement (mRSS decrease)
Elhai et al., 2013 [119]	27	Polyarthritis Myopathy mRSS	5 months	joint/muscle damage improvement skin thickening improvement (mRSS decrease)
Khanna et al., 2016 [114]	87	mRSS FVC	6 months	skin thickening improvement (mRSS decrease) smaller decrease in FVC
Khanna et al., 2017 [122]	78	mRSS FVC	24 months	skin thickening improvement (mRSS decrease) lung function stabilization
Khanna et al., 2018 [115]	210	mRSS FVC	12 months	skin thickening improvement (mRSS decrease) lung function stabilization
Denton et al., 2018 [116]	12	mRSS	6 months	skin thickening improvement (mRSS decrease) decreased skin collagen fiber production
Zacay et al., 2018 [118]	16	Arthritis/arthralgia Myalgia/myositis FVC mRSS	8 months	significant improvement in arthritis/arthralgia muscle enzymes normalization skin thickening improvement (mRSS decrease) lung capacity improvement (FVC increase)
Shima et al., 2019 [63]	7	mRSS	6 months	skin thickening improvement (mRSS decrease) especially in cases with significant inflammatory syndrome and short disease duration
Narvaez et al., 2019 [124]	9	FVC DLCO 6 min walking test	12 months	lung function stabilization and improvement (FVC increase) in cases refractory to corticosteroids or other immunosuppressants
Khanna et al. 2020 [121]	78	FVC	24 months	lung function stabilization / improvement (FVC increase)
Roofeh et al., 2021 [123]	136	FVC	12 months	lung function preservation according to the degree of pulmonary fibrosis
Khanna et al., 2021 [125]	82	mRSS FVC	24 months	skin thickening improvement (mRSS decrease) lung function improvement (FVC increase)
Adrovic et al., 2021 [130]	9	Juvenile SS mRSS DLCO	24 months	skin thickening improvement (mRSS decrease) lung function improvement (DLCO increase)
Panopoulos et al., 2022 [126]	21	mRSS Polyarthritis FVC PROs	12 months	skin thickening improvement (mRSS decrease) lung function stabilization joint damage improvement (Disease Activity Score 28 decrease) improving patients’ quality of life

Key: mRSS: modified RODNAN skin score; DLCO: diffusing capacity of lung for carbon monoxide; FVC: forced vital capacity; PROs: patient-reported outcomes.

## Data Availability

Not applicable.
